# Homozygous Sickle Cell Disease after Age of 40: Follow-Up of a Cohort of 209 Patients in Senegal, West Africa

**DOI:** 10.1155/2024/7501577

**Published:** 2024-02-06

**Authors:** Moussa Seck, Maureen Adéniké Dabo, Elimane Seydi Bousso, Mohamed Keita, Sokhna Aïssatou Touré, Sérigne Mourtalla Guèye, Blaise Félix Faye, Fatma Dieng, Saliou Diop

**Affiliations:** ^1^Hematology Department, Cheikh Anta Diop University of Dakar, Dakar, Senegal; ^2^National Blood Transfusion Center of Dakar, BP 5002, Fann, Dakar, Senegal

## Abstract

**Objectives:**

The aim of this study was to describe the morbidity and mortality of homozygous sickle cell disease after the age of 40.

**Methods:**

This was a cohort study of 209 patients followed from 1994 to 2022. All hemoglobin electrophoresis-confirmed SS sickle cell patients over 40 years were included. A descriptive study of epidemiological, diagnostic, therapeutic, and evolutionary data was used to assess morbidity and mortality.

**Results:**

Sex ratio (M/F) was 0.6. Median age was 47 (41–75). According to morbidity, 95.1% had less than 3 vaso-occlusive crises/year. Acute anemia was the most frequent complication (52.63%). Chronic complications were noted in 32.5%. At diagnosis, mean hemoglobin was 8.1 g/dl ± 1.9, HbS was 86.5 ± 10, and HbF was 9.4 ± 7.6. Number of patients transfused was 66%. We noted that 8.1% of patients died, 29.2% were lost to follow-up, and 62.7% were still being followed up. The risk factors identified for death were geographical origin, comorbidity, high HbS, low HbF, and thrombocytosis.

**Conclusion:**

This study shows that homozygous SCD is increasingly becoming an adult disease and that it can be carried into old age in Africa. Advanced age over 40 is marked by an upsurge in chronic complications, making it essential to set up a screening program and to organize multidisciplinary follow-up.

## 1. Introduction

Sickle cell disease (SCD) is one of the most common severe monogenic hereditary diseases in the world [1]. It affects more than 50 million people worldwide, including 38 million in sub-Saharan Africa [2], with 8 to 10% of Senegalese carriers of hemoglobin S [3]. Its frequency and severity make it a major public health problem worldwide. It is associated with early adult mortality and severe organic complications [4, 5]. According to the systematic analysis of the Global Burden of Disease Study, 176,000 people die every year from complications linked to sickle cell disease [6].

Currently, life expectancy is estimated at over 50 years in the United States but less than 10 years in sub-Saharan Africa [7]. With the advent of neonatal screening and new means of therapeutic management, patients' quality of life has improved, and it is not uncommon today to have patients between 50 and 70 years of age [8, 9]. However, SCD in adults evolves into a chronic multivisceral degenerative disease requiring multidisciplinary management involving several specialties [10].

In 1994, an American study of mortality in SCD patients found an average age of death of 42 for men and 48 for women [11]. The life expectancy of SCD sufferers is still more than 2 decades shorter than the general population average [12, 13].

In 2003, in sub-Saharan Africa, a study of homozygous SCD over 20 years of age showed that homozygous SCD could be lived with well into old age in Africa when patients benefited from regular follow-up. He also described the respective frequency of the various complications and the need to set up multidisciplinary teams to optimize care conditions in Africa [14]. Twenty years on, we decided to repeat this work on a larger cohort of older patients. Thus, in addition to assessing the morbidity and mortality of SS sickle cell disease in these patients aged over 40, we are going to carry out a descriptive study of the epidemiological, diagnostic, therapeutic, and evolutionary aspects.

## 2. Materials and Methods

The clinical haematology department at the National Blood Transfusion Center in Dakar, Senegal, is a reference center for the management of SCD patients. The follow-up care was set up in the 1990s. Around 3,000 major SCD patients are regularly monitored, with at least one consultation every 6 months. A census of this cohort revealed 209 homozygous SCD patients over 40 years of age, with a medical file containing full information (civil status, diagnostic, therapeutic, and evolutionary parameters).

Medical care for SCD in Senegal is entirely self-pay in adulthood. Hydroxyurea and folic acid are paid for entirely by the sickle cell patient. Blood transfusions in Senegal are free for patients and fully covered by the Ministry of Health.

We realized a cohort study of 209 homozygous SCD patients over 40 years of age followed from 1994 to 2022. SCD diagnosis was made on hemoglobin electrophoresis (SS profile: HbS => 80%, HbF < 15%, and HbA2 < 3%). We studied the following parameters:

1. *Basic Characteristics of Patients*. Age, sex, professional activities, marital status, the origin of patient, regularity of follow-up (defined as at least two consultations per year), duration of follow-up (time between diagnosis and death), presence of comorbidity (arterial hypertension, diabetes, asthma, and heart disease), baseline hemoglobin level, haemogram data, electrophoretic data, therapeutic aspects (hydroxyurea and transfusion program), prevention of anemia (folic acid), vaso-occlusive crisis (VOCs) prevention (therapeutic education and avoidance of triggers), and infection prevention (antibiotic therapy and vaccination).
2. *Morbidity Related to SCD*. Age at diagnosis, age of first clinical manifestation, number of VOCs per year, frequency of hospitalization, frequency of acute and chronic complications, and history of blood transfusion (number of patients transfused, transfusion indications, modalities, and complications).
3. *Assessing Mortality Related to SCD*. Number of patients who died, number of patients who died per year, causes of death, and risk factors for death. For patients who died while hospitalized on the ward, death details were recorded on the patient's file and filed in the death archive. In the case of patients who died at home, an interview with their family members enabled us to collect data relating to the death.
4. *Statistical Analysis*. Data were analyzed using the R foundation for statistical computing version 4.2.3. A descriptive section in which qualitative variables were described in terms of numbers and frequency, and quantitative variables in terms of means, standard deviation (SD), and maximum and minimum ages. The Student's *t* test was used for comparison between variables, the difference being significant when the p value was strictly less than 0.05 (*P* < 0.05).

## 3. Results

### 3.1. Basic Characteristics of SCD Patients

A total of 209 homozygous SCD over 40 years of age out of 2,453 SCD patients monitored were included in the study, representing a prevalence of 8.52%.

According to the biological diagnosis, the mean HbS level was 86.5% ± 10, the mean HbF level was 9.4 g/dl ± 7.6, and the mean HbA2 level was 2.9 g/dl ± 1.9. The mean baseline hemoglobin was 8.2 g/dl ± 1.4; the mean white blood cell count was 12.617 G/L ± 6.464, and the mean platelet count was 395.167 G/L ± 163.075.

**Figure 1:**
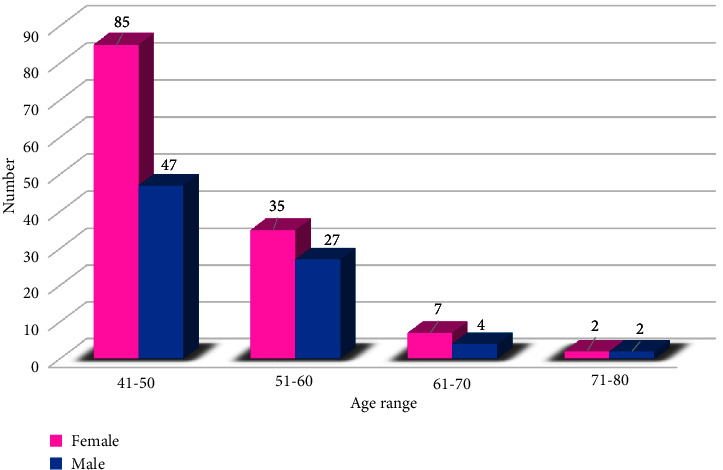
Distribution of sickle cell patients by age and gender.

The mean age was 49 ± 6.9, and the median age was 47 (41–75). The highest proportion of patients was in the 41–50 age group (62.68%). The sex ratio (M/F) was 0.6. The mean age was 48.9 ± 6.9 for women and 49.2 ± 6.9 for men (Figure 1). We noted that 51.9% of patients were not yet married and 89% of patients had a professional activity (there are patients with income-generating activity). The majority of patients were from Dakar (the capital of Senegal) (73.21%). More than 2/3 of patients (72.2%) had irregular follow-up (these are patients who do not come for a routine consultation at least every 6 months). We found that 77.6% of patients had no comorbidities. Immunization status was up to date in only 12.9% of patients. Almost all patients were receiving folic acid (99%), and only 1.43% were on hydroxyurea (Table 1).

### 3.2. Morbidity Related to SCD

The mean age at diagnosis was 3.1 ± 1.5 years. The mean age of onset of the first clinical manifestation was 11.1 years ± 11. More than 3/4 (95.1%) of patients had less than 3 VOCs/year. The average number of hospitalizations per year was 3.5 ± 2.8. The number of patients transfused was 66%. Simple transfusion was performed in 87.68%, and exchange transfusion was performed in 19.56%. The average number of transfusions per patient was 2.3 ± 2.9. A total of 164 transfusion procedures were performed. Acute anemia was the most frequent transfusion indication (50.6%), followed by prolonged VOCs (13.41%) and acute chest syndrome (12.8%) (Table 2).

Acute anemia was the most frequent acute complication (52.63%) followed by the acute chest syndrome (10%). Chronic complications were present in 32.5%, dominated by femoral head aseptic osteonecrosis (22.96%) and biliary lithiasis (16.74%) (Table 3).

According to gynecological and obstetrical history, the number of patients who had a pregnancy represented 66.1%; the average number of pregnancies was 2.4 ± 2.2; the average number of deliveries was 1.3 ± 1.5; the average number of abortions was 1.1 ± 1.8. Only 17.1% had undergone a caesarean section at the time of delivery (Table 4).

### 3.3. Assessing Mortality Related to SCD

Seventeen patients had died (8.1%), 62.7% were alive and being followed up, and 29.2% had been lost to follow-up (patients absent from consultations for more than 2 years). All epidemiological, diagnostic, therapeutic, and evolutionary factors that could be associated with patient death were analyzed. Only the factors listed in Table 5 were found to be associated with patient death.

## 4. Discussion

The frequency of SCD patients aged over 40 in our cohort was 8.52% (209/2453). We note that less than 10% of our patients are not yet 40 years of age. Several hypotheses can be put forward and will be the subject of future studies. It may be due to the fact that adult SCD patients no longer comply with regular follow-up and rarely attend medical appointments; on the other hand, due to the construction of new hospitals, many patients have changed follow-up departments, but it may also be linked to mortality, which is higher at this age. The highest age of patients in our cohort was 75 years. This shows that SCD can be experienced well into old age in Africa, although other studies in Africa show a maximum age below that found in our cohort [15, 16].

The most representative age group was between 41 and 50 and represented 62.68% of the population. This corresponds to a narrow-topped pyramid, as only 1.44% of patients were over 70. The mean age of patients was comparable according to gender, at 48.9 ± 6.9 for women and 49.2 ± 6.9 for men. Women predominated at 61.7%, with a sex ratio of 0.6. This female predominance was also observed by other authors in Africa who carried out studies among adult SCD patients [13, 17]. Less than a quarter of patients (11%) had no professional activity. This low rate of nonworking patients at this age has already been highlighted in other studies and mainly concerned women [18].

We found that few patients attended medical appointments regularly, with at least two visits per year. This has been observed in adult sickle cell cohorts, where patients of this age often neglect medical appointments. This can be explained by the rarity of VOCs at this stage of life and the patient's control of triggering factors [3, 15, 18].

From a therapeutic point of view, less than 1% of patients in our cohort were on hydroxyurea. The introduction of hydroxyurea in SCD treatment is still limited in Africa [15, 19]. In the multicenter study of SCD in sub-Saharan Africa, the same proportion was observed [20], which differs from developed countries, where proportions of around 64.5% of patients on hydroxyurea are reported [21]. This can be explained by many adverse effects of hydroxyurea, but also and above all by the unavailability and accessibility of the drug, given its high cost, beyond the reach of patients in Africa.

We noted a delay in the diagnosis of our patients. The average age of onset of the first clinical manifestation was 11.1 ± 11.3 years, which could explain this late diagnosis. The lesser severity of SCD in the Senegal haplotype could explain this delay in both clinical manifestations and biological diagnosis. Other studies in Africa have not shown this prolonged delay in diagnosis, but these studies have included SCD patients of all ages [19, 20, 22]. Biological confirmation of the diagnosis by electrophoretic testing was also delayed, as described in previous studies in the same department [23]. This delay in diagnosis and treatment may be explained by the absence of neonatal screening in our country.

SCD was discovered in most cases in the setting of VOCs (30.1%) but was also discovered incidentally in 17.1% of cases (during pregnancy follow-up or other etiological tests) or in the setting of chronic complications (6.2%). The lower clinical severity of SCD in our cohort meant that the proportion of patients with less than 3 VOCs/year was 95.1%. Other African countries reported lower rates, reflecting the severe morbidity of other SCD haplotypes [15, 24, 25].

In a study of transfusion practices among SCD patients in our country, simple transfusion was performed in 92.81% and exchange transfusion in 18.95% [26]. Our study confirms this finding, showing that simple transfusion was most frequently used. We also show that our patients received fewer transfusions compared with other countries in Africa and the West [12, 27, 28]. This lower frequency of transfusion in our cohort is due to the fact that our patients are not on a transfusion program, and the main indication for transfusion was worsening anemia.

The management of SCD relies on the prevention and treatment of SCD complications and is mainly supportive. Disease-modifying therapies for patients with SCD include chronic transfusion programs, hydroxyurea, and more recently, L-glutamine, crizanlizumab in 2021, and voxelotor in 2022. Potentially curative therapy includes hematopoietic stem cell transplantation and gene therapies. SCD has a significant negative impact on the quality of life of patients and requires high levels of healthcare resource utilization, resulting in a significant economic burden [5].

We have shown in this study that approximately 1% of our patients are on hydroxyurea therapy. It is well known that hydroxyurea improves morbidity and mortality in SCD patients [9, 13]. Since only a tiny proportion of our patients are on hydroxyurea, it would be very difficult to make a comparison with other studies with larger cohorts. Perhaps, the absence of hydroxyurea was the reason for the small number of patients over 40 years of age (209 out of a total of 2453 patients). Our country's current policy in the management of SCD patients is to make hydroxyurea available so that its prescription can be extended. A future study would be useful to better understand the effect of hydroxyurea on morbidity and mortality in our cohort.

According to gynecological history, we noted an average of 2.4 pregnancies for 1.3 deliveries; therefore, an average of 1.1 pregnancies did not reach term, with the occurrence of an abortion or stillbirth. This rate of interrupted pregnancies not reaching term has already been reported in women with sickle cell disease [15]. Strict, well-coordinated management by haematologists and gynaecologists-obstetricians is essential for monitoring these SCD women, whose pregnancies carry a high risk of fetal and maternal mortality.

The prevalence of chronic complications was 32.5% lower in the department's previous studies covering all ages [14]. This confirms the recrudescence of chronic complications in SCD patients in adulthood. Aseptic osteonecrosis was the most frequent chronic complication, followed by biliary lithiasis. These results are in line with the literature, where these two types of complication are more frequently described [3]. Most of these studies underestimate the true prevalence of these chronic complications, due to their often asymptomatic nature and the fact that, for reasons of cost and accessibility, they are not systematically screened.

The high proportion of patients lost to follow-up can be explained by age. Some patients prefer to be cared for at home, believing they know their disease better. The asymptomatic aspect of chronic complications represents a risk for some patients, who leave their medical monitoring altogether.

The death rate in our cohort was 8.1%. In our country, between 2011 and 2020, mortality among SCD patients of all ages was 2.76% [29]. This SCD mortality rate has improved significantly in recent years and is often correlated with the level of development in different countries [30]. Risk factors associated with patient death were identified. These included geographical origin, the presence of comorbidity, high HbS, low HbF, and the existence of thrombocytosis. HbF levels have been shown in previous studies to be a protective factor against morbidity and mortality in SCD patients [14, 31].

Extended life expectancy for SCD patients depends on the level of development of countries according to a well-coordinated organization. The earlier care is provided, and the more governments support it through free care or health insurance systems, the lower the mortality rate, and the longer the life expectancy of sickle cell patients [9, 13].

## 5. Conclusion

This study shows that homozygous SCID is increasingly becoming an adult disease and that it can be carried into old age in Africa. Advanced age over 40 is marked by an upsurge in chronic complications, making it essential to set up a screening program for these complications and to organize multidisciplinary follow-up.

We have also highlighted the irregularity of follow-up from this age onwards, which constitutes a risk for patients and a failure to detect chronic organic complications early. A comparative study with patients under 40 would enable us to better assess the severity of morbidity and mortality associated with sickle cell disease in patients over 40.

## Figures and Tables

**Table 1:** Basic characteristics of SCD patients.

Parameters	Patients over 40 (*n* = 209)	Patients under 40 (*n* = 2244)	*P*
Origin of patients			
Dakar (Senegalese capital)	153 (73.2%)	1688 (75.2%)	0.010
Others (Senegalese regions or country)	56 (26.8%)	556 (24.8%)	
Sex (sex ratio = 0.6)			
Female	130 (62.2%)	1164 (51.9%)	0.031
Male	79 (37.8%)	1079 (48.1%)	
Marital status			
Married	108 (51.79%)	727 (32.4%)	0.013
Unmarried	94 (44.9%)	1263 (56.3%)	
Divorced	7 (3.4%)	253 (11.3%)	
Patients with professional activity	186 (88.9%)	525 (23.4%)	0.001
Patients with regular follow-up	58 (27.8%)	1855 (82.7%)	0.010
Patients with comorbidities	47 (22.4%)	219 (9.7%)	0.001
Current vaccination status	27 (12.9%)	1283 (57.2%)	0.030
Therapeutic aspects			
Folic acid	206 (99%)	2212 (98.6%)	0.001
Hydroxyurea	3 (1.43%)	34 (1.51%)	0.020

**Table 2:** Morbidity related to SCD: frequency of VOCs, history of hospitalization, and transfusion practice.

Parameters	Number (*n* = 209)	Frequency (%)
Frequency of VOCs		
<3 VOCs/year	194	92.83
3–5 VOCs/year	07	3.35
>5 VOCs/year	08	3.82
Patients transfused	138	66.00
Blood transfusion procedures		
Single transfusion	121	57.80
Exchange transfusion	27	12.90
Indications for transfusion (*n* = 164)		
Acute anemia	83	50.60
Prolonged VOCs	22	13.41
Acute chest syndrome	21	12.80
Pregnancy	13	7.93
Preparation for surgery	11	6.72
Acute priapism	06	3.66
Leg ulcers	08	4.88

**Table 3:** Morbidity related to SCD: frequencies and types of acute and chronic complications.

Parameters	Numbers (*n* = 209)	Frequency (%)
Acute complications		
Acute anemia	110	52.63
Stroke	04	1.90
Acute chest syndrome	21	10.00
Priapism	11	5.26
Acute osteomyelitis	03	1.43
Frequency of chronic complications	68	32.50
Type of chronic complications		
Osteonecrosis	48	22.96
Biliary lithiasis	35	16.74
Leg ulcers	12	5.74
Nephropathy	16	7.65
Heart failure	09	4.30
Retinopathy	09	4.30
Pulmonary hypertension	08	3.82

**Table 4:** Morbidity related to SCD: gynecological and obstetrical data for SCD women.

Parameters	Average number (±SD)	Maximum	Minimum
Gestity	2.4 ± 2.2	10	1
Parity	1.3 ± 1.5	5	0
Abortion	1.1 ± 1.8	5	0
Stillbirths	1.3 ± 1.7	5	0
Simple transfusion	6.4 ± 6.2	20	1
Exchange transfusion	4	11	0

**Table 5:** Risk factors for death in SCD patients.

Parameters	Deceased patients (*n* = 17)	Living patients (*n* = 131)	*P*
Origin			
Dakar	5 (29.4%)	70 (53.4%)	0.049
Others	12 (70.6%)	61 (46.6%)	
Comorbidities	4 (23.5%)	18 (13.7%)	0.008
Mean HbS rate	90.2 ± 5.9	86.2 ± 10.2	0.019
Mean HbF rate	6.2 ± 6.0	9.7 ± 7.7	0.035
Mean platelet count	356900 ± 90864.3	394239.2 ± 165565.1	0.023

## Data Availability

The data used to support the conclusions of this study are available on request from the corresponding author.

## Additional Points

*Information*. (1) Original work describing for the first time the profile of homozygous sickle cell patients at advanced age beyond 40 years. (2) Advanced age is characterized by irregular follow-up, a recrudescence of chronic complications, and a low mortality rate. (3) We have shown that SS sickle cell disease can be lived with well into old age in Africa, despite the precarious and inadequate means of care.
